# Supplementary material for: Identification of photoperiod-induced specific miRNAs in the adrenal glands of Sunite sheep (Ovis aries)
Source: Front Vet Sci. 2022 Jul 22;9:888207. doi: 10.3389/fvets.2022.888207 (PMC9354845; doi:10.3389/fvets.2022.888207)
Supplement: Supplementary file 1 [file Data_Sheet_1.ZIP › Supplementary file/miRDeep2 file.zip!/miRDeep2 file/Novel_Structure/SP42A2/Novel_628.pdf]

| | | |
|---|---|---|
| Provisional ID | : | NC_019460.2_11533 |
| Score total | : | 43.6 |
| Score for star read(s) | : | 3.9 |
| Score for read counts | : | 36.8 |
| Score for mfe | : | 1.2 |
| Score for randfold | : | 1.6 |
| Score for cons. seed | : | |
| Total read count | : | 84 |
| Mature read count | : | 83 |
| Loop read count | : | 0 |
| Star read count | : | 1 |

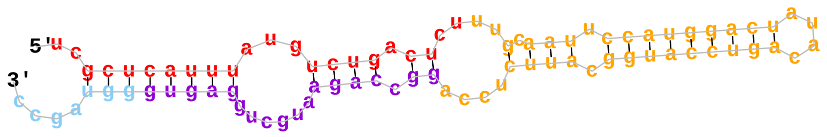


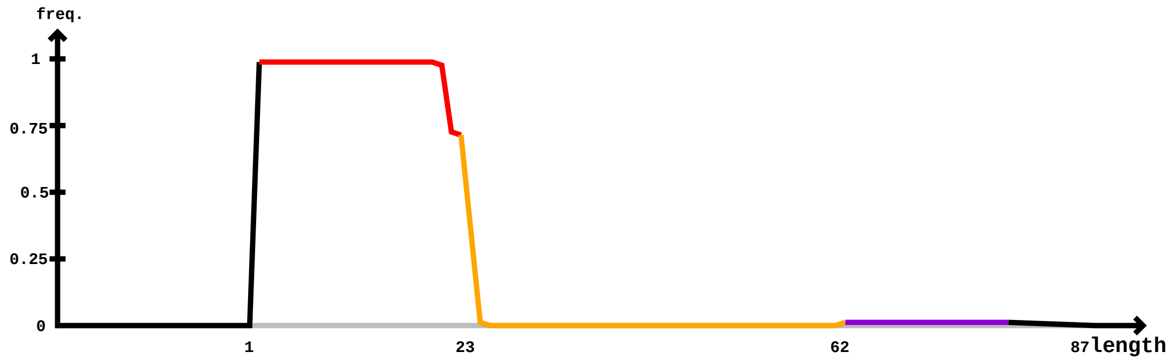


**Mature** **Star**

```
5'- caugaagugaaagugaaggucgcucauuuaugucugacucuuugcaauuccauggacuauacaguccauggcauucuccaggccagaaugcuggagugggguagccuuuccc   -3'    obs
    caugaagugaaagugaaggucgcucauuuaugucugacucuuugcaauuccauggacuauacaguccauggcauucuccaggccagaaugcuggagugggguagccuuuccc          exp
    .............((((((.((((((((...((((.((....(.(((.(((((((((....))))))))).))))....)).))))......)))))))).))))))...   reads    mm    sample
    ...................ucgcucauuCaugucugac..........................................................................   1        1     seq
    ...................ucgcucauuCaugucugacu.........................................................................   21       1     seq
    ...................ucgcucauuCaugucugacuc........................................................................   1        1     seq
    ...................ucgcucauuCaugucugacucu.......................................................................   56       1     seq
    ...................ucgcucauuCaugucugacucuu......................................................................   3        1     seq
    ...................ucgcucauuCaugucugacucuuu.....................................................................   1        1     seq
    ................................................................................ggcUagaaugcuggagug.............   1        1     seq
```
